# Muscle wasting and sarcopenia in heart failure: a brief overview of the current literature

**DOI:** 10.1002/ehf2.12388

**Published:** 2018-12-20

**Authors:** Stephan von Haehling

**Affiliations:** ^1^ Department of Cardiology and Pneumology University of Göttingen Medical Center Göttingen Germany; ^2^ Deutsches Zentrum für Herz‐und Kreislaufforschung, Standort Göttingen Göttingen Germany

## Introduction

Heart failure (HF) remains a major challenge in Western countries with up to 2% of the population being affected. Co‐morbidities have a huge impact on the clinical course and on patient outcomes. Such co‐morbidities include coronary artery disease, arterial hypertension, diabetes mellitus, chronic obstructive pulmonary disease (COPD), and chronic kidney disease (CKD). In recent years, wasting disorders have received increasing attention in patients with HF. These wasting disorders embrace involuntary loss of body weight—a condition that has been termed cardiac cachexia—as well as muscle wasting, also known as sarcopenia.

The French physician Charles Mauriac was the first to use the term cardiac cachexia in his medical dissertation that was published in 1860.^1^ The term cachexia, however, was introduced probably as early as during ancient Greek times because some sources claim that Hippocrates used the term already.^1^ The term sarcopenia, on the other hand, was coined in 1989 by Rosenberg and colleagues.^2^ It is important to understand the differences between the two types of wasting: cardiac cachexia develops in the course of manifest HF, which has progressed already to a comparatively late stage, implying that a catabolic state has already developed. Cachexia is frequent not only in patients with HF but also in patients with CKD, COPD, and neurological diseases as well as in rheumatoid arthritis.^3^, ^4^, ^5^ The diagnosis can be made by using weighing scale and by asking the patient for his or her weight before HF developed or 12 months before the actual date. In 1997, the presence of cardiac cachexia had been established as an independent risk factor for mortality in ambulatory patients with HF.^6^ Sarcopenia, on the other hand, was first identified as a co‐morbidity in patients with HF in 2013.^7^ Therefore, the guidelines of the European Society of Cardiology have started to mention cachexia and sarcopenia as relevant co‐morbidities of HF only in 2016. It was also in 2016 that sarcopenia received an International Classification of Diseases code, which was perceived as a major step forward in recognizing sarcopenia as a disease.^8^, ^9^

The original description of sarcopenia in HF used a rather heterogeneous cohort of ambulatory patients with HF including those with HF with reduced as well as those with HF with preserved ejection fraction. The prevalence of sarcopenia (muscle wasting) in that study was 19.5% of all patients included, and sarcopenia was an independent predictor of poor exercise capacity.^9^ Subsequent studies restricted to patients with HF with preserved ejection fraction have confirmed a similar prevalence.^10^ Whilst patients with sarcopenia primarily lose skeletal muscle, those who have cachexia may lose any type of tissue that ultimately yields weight loss. Recent data have confirmed that patients with muscle wasting in cachexia are particularly affected by reduced exercise capacity, strength, and quality of life.^11^

Treating wasting in patients with HF remains challenging. Approaches have included nutritional supplements,^12^, ^13^ exercise training,^14^ and drug treatments.^15^ The aim of this overview is to summarize the available knowledge of muscle wasting and sarcopenia in patients with HF. Most of the evidence is currently available from geriatric populations, and data from patients with HF remain rare.

## Prevalence and incidence of sarcopenia in elderly populations

The Newcastle 85+ Study recruited two waves of subjects born in 1921 and registered in Newcastle general practices.^16^ The authors of this study used the definition proposed by the European Working Group of Sarcopenia in Older People. This is one among the many different definitions of sarcopenia that have been suggested; however, no international consensus has been arrived at in this question. The study authors assessed grip strength, gait speed, and lean mass and found that sarcopenia was present in 21% of this British cohort of very old healthy subjects. Whilst the total sample included 719 participants, incidence data were available for 302 participants with an incidence rate for sarcopenia of 3.7 cases per 100 person‐years at risk. Another study by Brown *et al*.^17^ used a population‐based cohort with data of 4425 older adults from the Third National Health and Nutrition Survey with muscle mass being quantified by bioimpedance analysis and muscle function by gait speed. The mean age of this population was 70.1 years, and the prevalence of sarcopenia was 36.5%. The authors found that sarcopenia is associated with an increased risk of all‐cause mortality in men and women and also with increased risk of sarcopenia‐specific mortality among women but not men, identifying sarcopenia particularly in an elderly female population as a risk factor for cardiovascular adverse events. Tyrovolas *et al*. used data from 18 363 people aged 65 years or older who participated in the Collaborative Research on Ageing in Europe survey that was conducted in Finland, Poland, and Spain as well as data from the World Health Organization Study on Global Ageing and Adult Health Survey conducted in China, Ghana, India, Mexico, Russia, and South Africa.^18^ The prevalence of sarcopenia ranged from 12.6% (Poland) to 17.5% (India). Sarcopenic obesity, that is, obese subjects with low muscle mass, was identified in 1.3% (India) up to 11% (Spain). Low physical activity was an independent predictor of sarcopenia prevalence and sarcopenic obesity in the overall sample. A similar study in only 394 participants from the multicentre Italian Study conducted by the Gruppo Lavoro Italiano Sarcopenia—Trattamento e Nutrizione (GLISTEN) in 12 acute care wards of university hospitals across Italy determined a prevalence of sarcopenia at hospital admission of 14.7% in a sample with a mean age of 79.6 ± 6.4 years. These authors used the European Working Group of Sarcopenia in Older People sarcopenia diagnostic criteria at discharge. They found that patients who developed sarcopenia spent on average of 5.1 days in bed compared with 3.2 days for those with no sarcopenia at discharge. This study identifies sarcopenia as an important factor to prolong hospital stay in patients admitted to acute care wards.^19^ Yang *et al*. conducted a prospective observational study in acute care wards in teaching hospitals in Western China and obtained the survival status and readmission information after 12, 24, and 36 months after the baseline investigation. Among 288 participants (mean age 81.1 ± 6.6 years), 49 were identified as having sarcopenia (17%). The prevalence was similar in men and women. During 3 years of follow‐up, 49 men and 9 women died. Sarcopenic patients were more likely to die than non‐sarcopenic patients, and after adjusting for age, sex, and other confounders, sarcopenia remained an independent predictor of 3‐year mortality and readmission.^20^ These data confirm that sarcopenia is very prevalent among elderly subjects and is an independent predictor of readmission and mortality in acute care wards and is a factor that increases the duration of hospital stay. In this context, it is important to highlight the importance of systemic inflammation in patients with sarcopenia and low physical performance, both at a pathophysiological and at a clinical level.^21^

Makizako and colleagues studied physical performance and body composition in community‐dwelling Japanese older adults using cross‐sectional data from 10 092 subjects analysing handgrip strength, the five‐times‐sit‐to‐stand test, and gait speed. These parameters of physical performance decreased significantly with ageing. The slope of decline in age‐associated changes was greater in grip strength for men and walking speed for women. Importantly, these authors found that the decrease in appendicular skeletal mass index associated with age is much more prominent than that of body mass index.^22^ Among elderly subjects from the UK, Zengin *et al*. studied men aged 40–79 years recruited from Manchester. They analysed lower limb jump force and power by measuring a single two‐leg jump performed on a ground reaction force platform. Grip strength on the other hand was measured using a handgrip dynamometer in a total of 301 men. They found that jump force is negatively associated with age. For every 10 year increase in age, grip strength decreased by 11%. Interestingly, jump force was positively associated with tibial bone outcomes.^23^ These findings buttress the loss in muscle strength with advancing age. They also serve as a basis for understanding the increased risk of falls as assessed in the STRAMBO study. These authors studied 890 men aged 50 years and older and assessed appendicular skeletal muscle mass by dual‐energy X‐ray absorptiometry (DEXA) scan, grip strength, and physical function. A total of 813 men aged 60 and older were followed out to 5 years. Overall, the authors found that low leisure physical activity, very high occupational physical activity, Parkinson's disease, diabetes mellitus, low apparent free testosterone concentration, and vertebral and multiple fractures were associated with lower grip strength. On the other hand, multiple vertebral fractures were associated with a two‐fold higher risk of multiple falls after adjustment for confounders.^24^ Another large study in 3493 non‐institutionalized older adults found that approximately 30% of the participants were at risk for losing physical independence at 90 years of age.^25^ The aforementioned data underline the fact that the loss of skeletal muscle mass is associated with an increased risk of morbidity and mortality and in particular with an increased risk of losing physical independence. Therefore, the maintenance of skeletal muscle mass should be regarded as paramount in all elderly subjects; however, the moment patients have manifest HF, the maintenance of skeletal muscle mass has to be taken to an even higher level of importance.

Interestingly, data from the Geelong Osteoporosis Study confirm these findings using data from a cohort of 750 women aged 50–94 years who were followed for a decade after femoral neck bone mineral density and an appendicular lean mass assessment by dual energy X‐ray absorptiometry (DEXA). These authors found that during 6712 person‐years of follow‐up, there were 190 deaths, and proportions were increasing with diminishing bone mineral density as well as with diminishing appendicular lean mass, the defining feature of sarcopenia. These authors conclude that poor musculoskeletal health increases the risk of mortality independent of age, an effect that appears to be driven mainly by a decline in bone mass. However, low lean mass independently exacerbates the mortality risk, and this effect appears to operate through poor health exposures.^26^ Indeed, using data from the Korean National Health and Nutrition Examination Survey, Kim *et al*. found that higher vitamin D levels were significantly associated with a reduced likelihood of ‘adverse body composition’. In particular, patients in the highest tertile of serum vitamin D levels were less likely to have osteosarcopenic obesity, whereas vitamin D sufficiency in men was significantly associated with in increased likelihood of a higher number of adverse body composition.^27^

## Screening and diagnosing sarcopenia

The pathophysiology of wasting in HF remains incompletely understood. This is also true for sarcopenia in healthy ageing. An anabolic–catabolic imbalance does certainly play a role as does an alteration in appetite and food intake. On the other hand, decreased exercise capacity and decreased physical activity act hand in hand in aggravating skeletal muscle loss, in itself accelerated by an imbalance in protein senescence and degradation.^28^, ^29^ Protein degradation is maintained via three pathophysiological avenues, which include the ubiquitin‐proteasome pathway, autophagy, and apoptosis mediated by caspase signalling.^30^ The human skeletal muscle proteome project is currently underway to characterize muscle proteins and the way they change during ageing and disease.^31^, ^32^ It has been established that patients with HF and sarcopenia have impaired endothelium function with lower vasodilation having a negative impact of exercise capacity, which is particularly prevalent in sarcopenic patients.^33^ Anorexia is likewise very prevalent in patients with HF as assessed using a 6‐point Likert scale.^34^ In patients with dilated cardiomyopathy, an even higher prevalence has been established. Hajahmadi *et al*. found in 55 patients with dilated cardiomyopathy that muscle wasting was prevalent in 47.3% of all patients. Patients with muscle wasting had lower left ventricular ejection fraction, lower 6 min walk distance, and a higher New York Heart Association functional class and hospitalization rate.^35^ In patients on the intensive care unit, muscular weakness and muscle wasting may be highly prevalent and may, for example, present as failure to wean from mechanical ventilation. On the other hand, mechanical ventilation itself may induce progressive dysfunction of the main respiratory muscle, that is, the diaphragm. Such problems may be particularly important in patients admitted to the intensive care unit with sarcopenia being already present.^36^ Cheung *et al*. recently reported that testosterone deprivation selectively decreases lower limb muscle function, predominantly affecting muscles that support body weight and muscles that accelerate the body forward during walking and mediate balance. Therefore, androgen deprivation appears to cause selective deficits in the biochemical leg muscle function, which may also play a role in patients with HF in whom the deficiency of anabolic hormones has been reported to play a major role and to have prognostic significance.^37^, ^38^ Anorexia, on the other hand, plays also a major role in the pathogenesis of wasting in both sarcopenia and cachexia.^39^ This goes hand in hand with the appearance of malnutrition. Intervention studies have mostly focused on *n*‐3 fatty acids; however, the clinical evidence to support a benefit of such nutritional interventions is still insufficient.^40^, ^41^

**Figure 1:**
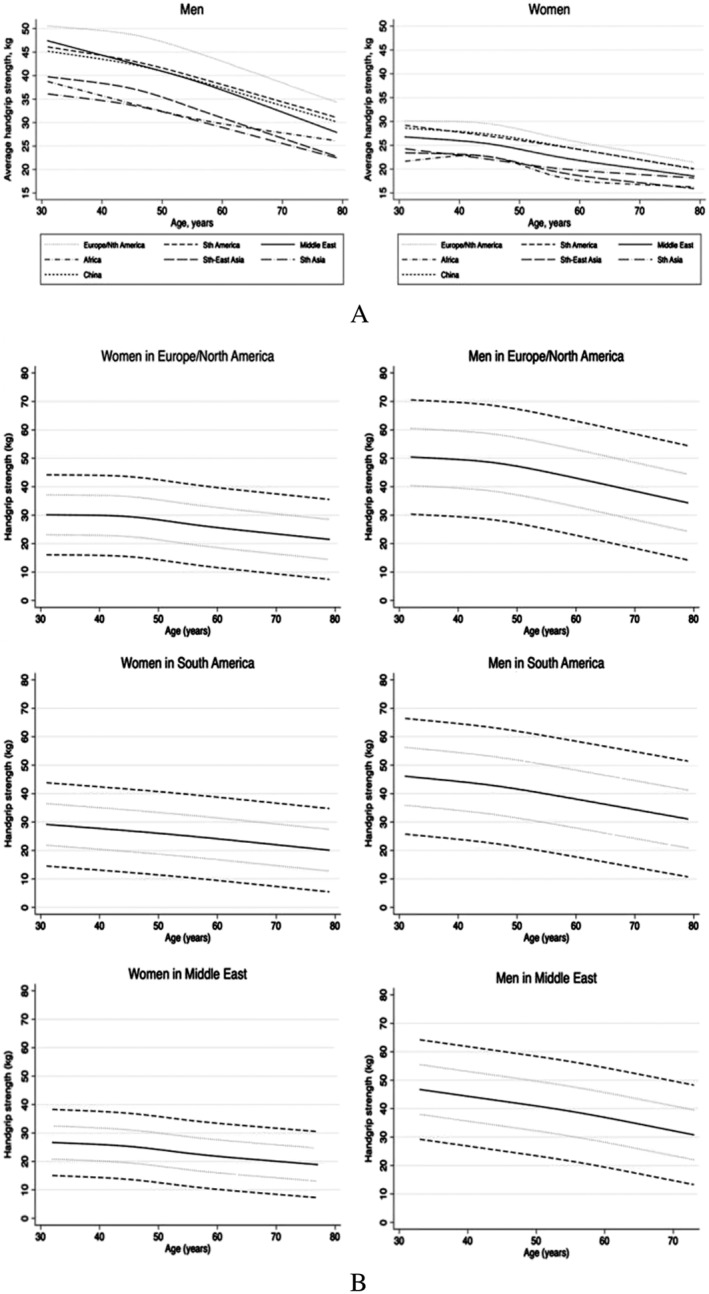
(A) Average handgrip strength as a function of age. Nth, North; Sth, South. Reproduced with permission from Leong *et al*.^54^ (B) Estimated handgrip strength (solid line) as a function of age. The dotted curves represent ±1 SD, and the dashed curves represent ±2 SD. Reproduced with permission from Leong *et al*.^54^

As discussed earlier, the clinical definition of sarcopenia remains a matter of debate, and a number of screening tools have been developed. SARC‐F, for example, is a symptom score that exhibits good internal consistency and may have value in the screening for sarcopenia.^42^ Very similarly, the SarQoL is a specific health‐related quality of life questionnaire for patients with sarcopenia. A recent study found that it is valid, consistent, and reliable and can be recommended for clinical and research purposes, even though its sensitivity to change needs to be assessed in additional studies.^43^ None of these screening tools have, however, been evaluated in the setting of HF. Other approaches have used the assessment of muscle quantity using ultrasound. Nijholt *et al*. found after analysing 17 studies that ultrasound is reliable and valid tool for the assessment of muscle size in older adults. On the other hand, these authors admit that more high‐quality research is required to confirm these findings in clinical and healthy populations and that ultrasound assessment requires more evaluation in smaller muscles. Scott and colleagues studied panoramic ultrasound and magnetic resonance imaging of the quadriceps and gastrocnemius muscles of the right leg in 27 subjects. They found that panoramic ultrasound imaging is a valid tool for monitoring quadriceps muscle atrophy and hypertrophy and for detecting gastrocnemius atrophy.^44^ The aforementioned screening tools and devices may help in screening patients for the presence for sarcopenia.^45^ However, the gold standard for the diagnosis remains the assessment using DEXA scan, computed tomography (CT), or magnetic resonance imaging. Biomarkers do not currently play a major role even though a large number of potential candidates has been evaluated.^46^, ^47^, ^48^, ^49^ An interesting candidate biomarker C‐terminal agrin fragment has been used in patients with HF^50^ and in patients after acute stroke.^51^ Sensitivity and specificity of biomarkers such as C‐terminal agrin fragment and other such candidates remain however unsatisfying.^52^ Another interesting approach is the assessment of handgrip strength using a handgrip dynamometer. The debate is ongoing on how many attempts are needed to assess the maximum handgrip strength in order to identify individuals with low muscle strength, and it might in fact be the case that the number of attempts may have an influence in this regard. Reihnierse and colleagues studied three cohorts including a total of 939 individuals and assessed the handgrip strength three times.^53^ Interestingly, the results showed the same pattern in all three cohorts with the maximal handgrip strength at Attempts 1 and 2 being higher than Attempt 3. Overall, values were highly reproducible. The authors thus concluded that maximal handgrip strength is dependent on the number of attempts but independent of age and health status. To assess maximal handgrip strength, at least three attempts should be made when handgrip strength is considered as a continuous valuable. If handgrip strength is, however, considered as discrete variable to assess low muscle strength, two attempts may be sufficient in younger populations. This point is important because single assessments may yield misclassifications of patients and may not prompt accurate diagnostic workups. Leong and colleagues established references ranges of handgrip strength in using data assessed by Jamar dynamometer in 125 462 healthy adults aged 35–70 years from 21 countries.^54^ Their findings were summarized in the Prospective Urban Rural Epidemiologic Study and found that handgrip strength values differed among individuals from different geographical regions. Values were the highest in Europe and North America and lowest in South Asia, Southeast Asia, and Africa and intermediate among patients or subjects from China, South America, and the Middle East (*Figure* 1). This point has to be considered when assessing handgrip strength in subjects from different geographical regions.

Kohara and colleagues study two sarcopenic indices: thigh muscle cross‐sectional area calculated by CT imaging and skeletal muscle mass assessed by bioelectric impedance. They defined muscle mass decline as either the bottom 10 or 20% of participants for each sex even though that methodological approach may have its pitfalls. It is interesting to note that lower sarcopenic indices were significantly related to lower cognitive scores. The authors assume that arterial stiffness and white matter hyperintensities could at least in part account for this association.^55^ In the context of decreasing skeletal muscle performance, the role of mitochondria needs to be mentioned. Indeed, mitochondria play a central role as principal source of intracellular reactive oxygen species, which are mainly formed in the respiratory chain. Ageing organs have been associated with dysfunctional mitochondria that generate less adenosine triphosphate. Such ageing mitochondria may play a role in senescence, and this effect may be particularly important in tissues that require high energy and thus high mitochondrial content such as the heart and the skeletal muscle. Fabbri and colleagues studied 511 men and women aged 50 years or older who were followed for an average of 4 years. Using DEXA scan as well as knee‐extension isokinetic strength and CT imaging of thigh muscle cross‐sectional area, they found that muscle quality shows a significant linear decline over time of the follow‐up, an effect that is independent of covariates such as baseline body weight and body mass index. Instead, higher total fat mass and lower total lean mass at baseline predicted a steep longitudinal decline in muscle quality.^56^ In patients with HF, on the other hand, it is interesting to note that carvedilol attenuates the development of cachexia and promotes a partial reversal of cachexia in patients with severe chronic HF.^57^

## Physical exercise and rehabilitation

Physical exercise and exercise training are important therapeutic approaches in muscle wasting and sarcopenia. Foong *et al*. studied 636 community‐dwelling older adults with a mean age of 66 ± 7 years. The muscle mass was measured using DEXA scan, and lower limb strength was measured via dynamometry. Using accelerometer measurement, the authors found that physical activity intensity was positively associated with both lean mass percentage and lower limp strength in dose–response fashion. Sedentary activity, on the other hand, was negatively associated with lean mass percentage but not with lower limb strength. Therefore, accelerometer assessment provides a readily available tool to follow patients' physical activity.^58^ Lemos Pinto and colleagues performed a 12 week parallel‐group, double‐blind, randomized, controlled trial in which individuals were allocated into one of two groups. The first received placebo and resistance training, and the second received creatine supplementation and resistance training. Patients were assessed using DEXA scan at baseline and after 12 weeks at time points in which they also had their muscle strength assessed. Interestingly, the group that received creatine supplementation and resistance training had superior gains in limb mass compared with the placebo and resistance training group. Unfortunately, muscle strength did not differ significantly.^59^ A study in 146 women with sarcopenic obesity found that a 3 week weight‐reducing programme combined with aerobic exercise lead to a significant weight reduction. Interestingly, obese women without sarcopenia lost lean mass, which was not the case in women with sarcopenic obesity.^60^ A systematic review of exercise rehabilitation following intensive care unit discharge for recovery of critical illness found an overall disappointing quality of evidence. The review of six trials involves 483 patients, but only two studies evaluated health‐related quality of life, and neither of these reported differences between intervention and control group. Therefore, the authors concluded that additional studies are necessary after intensive care unit discharge.^61^ An interesting approach was presented by Chan *et al*. who performed a randomized controlled trial of integrated care for patients with frailty and sarcopenia: 289 patients were randomized to one of two groups. The first received a 2 h education course of frailty, sarcopenia, coping strategies, nutrition, and a demonstration of study exercise programmes. They also received educational multimedia material, and patients underwent bimonthly telephone follow‐ups. The second group received all of the above and six sessions of on‐site problems‐solving therapy and 48 exercise sessions within 6 months. In addition, a brief nutrition consultation was also provided. Mean age of the participants was 71.6 ± 4.4 years with 53% being female. The improvement of the primary outcome, the cardiovascular health study phenotypic classification of frailty index, was 65% at 3 months and increase to 40% at 6 months. There was no difference between the two groups.^62^ In the context of resistance training, it is important to note that muscle fibre capillarization may be a critical factor for allowing muscle fibre hypertrophy due to resistance exercise training,^63^ even though the coupling between skeletal muscle fibre size and capillarization is maintained during healthy ageing.^64^

## Co‐morbidities of heart failure

It is important to note that co‐morbidities of HF are frequently associated with the development of muscle wasting and/or cachexia just like HF itself. Chronic kidney disease, for example, is prevalent in approximately 50% of all patients with HF, and CKD itself has been associated with wasting, because, very similar to HF, inflammation is involved in mediating skeletal muscle wasting in these patients. Chronic kidney disease promotes muscle inflammation through an up‐regulation of toll‐like receptor 4, which may activate downward inflammatory signals such as tumour necrosis factor and nuclear factor κB‐regulated genes.^65^ Like in HF, it has been difficult to suppress muscle wasting in patients with CKD. *In vitro* and *in vivo* models have used, for example, dexamethasone administration as well as ursolic acid derived from plants. In this *in vivo* model, ursolic acid improved CKD‐induced muscle wasting by suppressing the expression of myostatin and inflammatory cytokines.^66^ Like in osteopenia development mentioned earlier, vitamin D acts a as a modulator of musculoskeletal health in patients with CKD.^67^ Indoxyl sulfate induces mitochondrial dysfunction in models with CKD. This uraemic toxin is known to accelerate skeletal muscle atrophy, and therefore, indoxyl sulfate may be a potential target for mitrochondrial‐targeted intervention in CKD‐induced muscle atrophy.^68^ In patients on haemodialysis, the valid detection of sarcopenia may be particularly difficult among obese subjects. Indeed, the valid detection requires adjustment for body size, because skeletal muscle mass normalized only for height may underestimate the prevalence of low muscle mass particularly in overweight and obese subjects on haemodialysis.^69^ Likewise, patients with COPD likewise frequently present with cachexia and wasting. Both may be partly reversible in patients with COPD; however, they affect disease progression and prognosis in an adverse manner.^70^ One possible approach is again exercise training, which has been proven to be effective in improving lower limb muscle strength and exercise performance in patients with COPD and moderate airflow obstruction. Again, nutritional supplements may have an additional effect on nutritional status, inspiratory muscle strength, and physical activity.^71^

## Conclusions

The pathophysiological understanding of wasting in patients with HF is improving; however, many questions remain unsolved. In geriatric populations, low physical activity is an independent predictor of sarcopenia prevalence and sarcopenic obesity. Moreover, sarcopenia is an important factor in prolonging hospital stay among patients admitted to acute care wards. The loss of skeletal muscle mass is associated with an increased risk of morbidity and mortality and in particular with an increased risk of losing physical independence. Therapeutic approaches include nutritional supplements, exercise, and possibly drug treatments, but evidence remains unsatisfying. The implementation of sarcopenia into the HF guidelines of the European Society of Cardiology and the fact that sarcopenia has now an International Statistical Classification of Diseases code were important steps forward. In HF, a lot remains to be performed in order to better characterize the pathophysiology and to establish treatments.

## Conflict of interest

None declared.

## References

[1] Doehner W , Anker SD . Cardiac cachexia in early literature: a review of research prior to Medline. Int J Cardiol 2002; 85: 7–14. 12163205 10.1016/s0167-5273(02)00230-9

[2] Roubenoff R , Heymsfield SB , Kehayias JJ , Cannon JG , Rosenberg IH . Standardization of nomenclature of body composition in weight loss. Am J Clin Nutr 1997; 66: 192–196. 9209192 10.1093/ajcn/66.1.192

[3] von Haehling S , Anker MS , Anker SD . Prevalence and clinical impact of cachexia in chronic illness in Europe, USA and Japan: facts and numbers update 2016. J Cachexia Sarcopenia Muscle 2016; 7: 507–509. 27891294 10.1002/jcsm.12167 PMC5114624

[4] Sanders KJ , Kneppers AE , van de Bool C , Langen RC , Schols AM . Cachexia in chronic obstructive pulmonary disease: new insights and therapeutic perspective. J Cachexia Sarcopenia Muscle 2016; 7: 5–22. 27066314 10.1002/jcsm.12062 PMC4799856

[5] Konishi M , Ishida J , Springer J , Anker SD , von Haehling S . Cachexia research in Japan: facts and numbers on prevalence, incidence and clinical impact. J Cachexia Sarcopenia Muscle 2016; 7: 515–519. 27239422 10.1002/jcsm.12117 PMC4864161

[6] Anker SD , Ponikowski P , Varney S , Chua TP , Clark AL , Webb‐Peploe KM , Harrington D , Kox WJ , Poole‐Wilson PA , Coats AJ . Wasting as independent risk factor for mortality in chronic heart failure. Lancet 1997; 349: 1050–1053. 9107242 10.1016/S0140-6736(96)07015-8

[7] Fülster S , Tacke M , Sandek A , Ebner N , Tschöpe C , Doehner W , Anker SD , von Haehling S . Muscle wasting in patients with chronic heart failure: results from the Studies Investigating Co‐morbidities Aggravating Heart Failure (SICA‐HF). Eur Heart J 2013; 34: 512–519. 23178647 10.1093/eurheartj/ehs381

[8] Anker SD , Morley JE , von Haehling S . Welcome to the ICD‐10 code for sarcopenia. J Cachexia Sarcopenia Muscle 2016; 7: 512–514. 27891296 10.1002/jcsm.12147 PMC5114626

[9] Morley JE , Anker SD . Myopenia and precision (P4) medicine. J Cachexia Sarcopenia Muscle 2017; 8: 857–863. 28944582 10.1002/jcsm.12231 PMC5700444

[10] Bekfani T , Pellicori P , Morris DA , Ebner N , Valentova M , Steinbeck L , Wachter R , Elsner S , Sliziuk V , Schefold JC , Sandek A , Doehner W , Cleland JG , Lainscak M , Anker SD , von Haehling S . Sarcopenia in patients with heart failure with preserved ejection fraction: impact on muscle strength, exercise capacity and quality of life. Int J Cardiol 2016; 222: 41–46. 27454614 10.1016/j.ijcard.2016.07.135

[11] Emami A , Saitoh M , Valentova M , Sandek A , Evertz R , Ebner N , Loncar G , Springer J , Doehner W , Lainscak M , Hasenfuß G , Anker SD , von Haehling S . Comparison of sarcopenia and cachexia in men with chronic heart failure: results from the Studies Investigating Co‐morbidities Aggravating Heart Failure (SICA‐HF). Eur J Heart Fail 2018; 20: 1580–1587. 30160804 10.1002/ejhf.1304

[12] Rozentryt P , von Haehling S , Lainscak M , Nowak JU , Kalantar‐Zadeh K , Polonski L , Anker SD . The effects of a high‐caloric protein‐rich oral nutritional supplement in patients with chronic heart failure and cachexia on quality of life, body composition, and inflammation markers: a randomized, double‐blind pilot study. J Cachexia Sarcopenia Muscle 2010; 1: 35–42. 21475692 10.1007/s13539-010-0008-0 PMC3060643

[13] Burden ST , Gibson DJ , Lal S , Hill J , Pilling M , Soop M , Ramesh A , Todd C . Pre‐operative oral nutritional supplementation with dietary advice versus dietary advice alone in weight‐losing patients with colorectal cancer: single‐blind randomized controlled trial. J Cachexia Sarcopenia Muscle 2017; 8: 437–446. 28052576 10.1002/jcsm.12170 PMC5476846

[14] von Haehling S , Ebner N , Dos Santos MR , Springer J , Anker SD . Muscle wasting and cachexia in heart failure: mechanisms and therapies. Nat Rev Cardiol 2017; 14: 323–341. 28436486 10.1038/nrcardio.2017.51

[15] von Haehling S . Casting the net broader to confirm our imaginations: the long road to treating wasting disorders. J Cachexia Sarcopenia Muscle 2017; 8: 870–880. 29168628 10.1002/jcsm.12256 PMC5700431

[16] Dodds RM , Granic A , Davies K , KIrkwood TBL , Jagger C , Sayer AA . Prevalence and incidence of sarcopenia in the very old: findings from the Newcastle 85+ Study. J Cachexia Sarcopenia Muscle 2017; 8: 329–337. 10.1002/jcsm.12157 PMC5377385 27897431

[17] Brown JC , Harhay MO , Harhy MN . Sarcopenia and mortality among a population‐based sample of community‐dwelling older adults. J Cachexia Sarcopenia Muscle 2016; 7: 290–298. 27239410 10.1002/jcsm.12073 PMC4864252

[18] Tyrovolas S , Koyanagi A , Olaya B , Ayoso‐Mateos JL , Chatterji S , Tobiasz‐Adamczyk B , Koskinen S , Leonardi M , Haro JM . Factors associated with skeletal muscle mass, sarcopenia, and sarcopenic obesity in older adults: a multi‐continent study. J Cachexia Sarcopenia Muscle 2016; 7: 312–321. 27239412 10.1002/jcsm.12076 PMC4864288

[19] Martone AM , Bianchi L , Abete P , Bellelli G , Bo M , Cherubini A , Corica F , Di Bari M , Maggio M , Manca GM , Marzetti E , Rizzo MR , Rossi A , Volpato S , Landi F , The GLISTEN Group Investigators . The incidence of sarcopenia among hospitalized older patients. Results from the Listen study. J Cachexia Sarcopenia Muscle 2017; 8: 907–914. 28913934 10.1002/jcsm.12224 PMC5700449

[20] Yang M , Hu X , Wang H , Zhang L , Hao Q , Dong B . Sarcopenia predicts readmission and mortality in elderly patients in acute care wards: a prospective study. J Cachexia Sarcopenia Muscle 2017; 8: 251–258. 27896949 10.1002/jcsm.12163 PMC5377397

[21] Calvani R , Marini F , Cesari M , Buford TW , Manini TD , Pahor M , Leeuwenburgh C , Vernabei R , Landi F , Marzetti E . Systemic inflammation, body composition, and physical performance in old community‐dwellers. J Cachexia Sarcopenia Muscle 2017; 8: 69–77. 27897412 10.1002/jcsm.12134 PMC5326820

[22] Makizako H , Shimada H , Doi T , Tsutsumimoto K , Lee S , Lee SC , Harada K , Hotta R , Nakakubo S , Bae S , Harada K , Yoshida D , Uemura K , Anan Y , Park H , Suzuki T . Age‐dependent changes in physical performance and body composition in community‐dwelling Japanese older adults. J Cachexia Sarcopenia Muscle 2017; 8: 607–614. 28597612 10.1002/jcsm.12197 PMC5566639

[23] Zengin A , Pye S , Cook MJ , Adams JE , Rawer R , Wu FCW , O'Neill TW , Ward KA . Associations of muscle force, power, cross‐sectional muscle area and bone geometry in older UK men. J Cachexia Sarcopenia Muscle 2017; 8: 598–606. 28474432 10.1002/jcsm.12198 PMC5566651

[24] Szulc P , Feyt C , Chapurlat R . High risk of fall, poor physical function, and low grip strength in men with fracture—the STRAMBO study. J Cachexia Sarcopenia Muscle 2016; 7: 299–311. 27239407 10.1002/jcsm.12066 PMC4864191

[25] Dos Santos L , Cyrino ES , Antunes M , Santos DA , Sarinha LB . Sarcopenia and physical independence in older adults: the independent and synergic role of muscle mass and muscle function. J Cachexia Sarcopenia Muscle 2017; 8: 245–250. 27897417 10.1002/jcsm.12160 PMC5377449

[26] Pasco JA , Mohebbi M , Holloway KL , Rennan‐Olsen SL , Hyde NK , Kotowicz MA . Musculoskeletal decline and mortality: prospective data from the Geelong Osteoporosis Study. J Cachexia Sarcopenia Muscle 2017; 8: 482–489. 28025860 10.1002/jcsm.12177 PMC5476862

[27] Kim J , Lee Y , Kye S , Shung YS , Lee O . Association of serum vitamin D with osteosarcopenic obesity: Korea National Health and Nutrition Examination Survey 2008–2010. J Cachexia Sarcopenia Muscle 2017; 8: 259–266. 27897409 10.1002/jcsm.12154 PMC5377393

[28] Okita K , Kinugawa S , Tsutsui H . Exercise intolerance in chronic heart failure‐skeletal muscle dysfunction and potential therapies. Circ J 2013; 77: 293–300. 23337207 10.1253/circj.cj-12-1235

[29] von Haehling S , Ebner N , Dos Santos MR , Springer J , Anker SD . Role of microRNAs in wasting in heart failure. Nat Rev Cardiol 2017; 14: 566. 10.1038/nrcardio.2017.123 28770867

[30] Glass DJ . Signaling pathways perturbing muscle mass. Curr Opin Clin Nutr MetabCare 2010; 13: 225–229. 10.1097/mco.0b013e32833862df 20397318

[31] Gonzalez‐Feire M , Semba RD , Ubaida‐Mohlen C , Fabbri E , Scalzo P , Hojlund K , Dufresne C , Lyashkov A , Ferruci L . The Human Skeletal Muscle Proteome Project: a reappraisal of the current literature. J Cachexia Sarcopenia Muscle 2017; 8: 5–18. 27897395 10.1002/jcsm.12121 PMC5326819

[32] Ebhardt HA , Degen S , Tadin V , Schilb A , Johns N , Greig CA , Fearon KCH , Aebersold R , Jacobi C . Comprehensive proteome analysis of human skeletal muscle in cachexia and sarcopenia: a pilot study. J Cachexia Sarcopenia Muscle 2017; 8: 567–582. 28296247 10.1002/jcsm.12188 PMC5566647

[33] Dos Santos MR , Saitoh M , Ebner N , Valentova M , Konishi M , Ishida J , Springer J , Sandek A , Doehner W , Anker SD , von Haehling S . Sarcopenia and Endothelial Function in Patients With Chronic Heart Failure: Results From the Studies Investigating Comorbidities Aggravating Heart Failure (SICA‐HF). J Am Med Dir Assoc 2017; 18: 240–245. 27816483 10.1016/j.jamda.2016.09.006

[34] Saitoh M , Dos Santos MR , Emami A , Ishida J , Ebner N , Valentova M , Bekfani T , Sandek A , Lainscak M , Doehner W , Anker SD , von Haehling S . Anorexia, functional capacity, and clinical outcome in patients with chronic heart failure: results from the Studies Investigating Co‐morbidities Aggravating Heart Failure (SICA‐HF). ESC Heart Fail 2017; 4: 448–457. 28960880 10.1002/ehf2.12209 PMC5695184

[35] Hajahmadi M , Shmeshadi S , Khalilipur E , Amin A , Taghavi S , Maleki M , Malek H , Naderi N . Muscle wasting in young patients with dilated cardiomyopathy. J Cachexia Sarcopenia Muscle 2017; 8: 542–548. 28251827 10.1002/jcsm.12193 PMC5566643

[36] Berger D , Bloechlinger S , von Haehling S , Doehner W , Takala J , Graggen WJ , Schefold JC . Dysfunction of respiratory muscles in critically ill patients on the intensive care unit. J Cachexia Sarcopenia Muscle 2016; 7: 403–412. 27030815 10.1002/jcsm.12108 PMC4788634

[37] Jankowska E . Circulation 2006.

[38] Cheung AS , Gray H , Schache AG , Hoermann R , Lim Joon D , Zajac JD , Pandy MG , Grossmann M . Androgen deprivation causes selective deficits in the biomechanical leg muscle function of men during walking: a prospective case–control study. J Cachexia Sarcopenia Muscle 2017; 8: 102–112. 27897410 10.1002/jcsm.12133 PMC5326829

[39] Morley JE . Anorexia of ageing: a key component in the pathogenesis of both sarcopenia and cachexia. J Cachexia Sarcopenia Muscle 2017; 8: 523–526. 28452130 10.1002/jcsm.12192 PMC5566640

[40] Konishi M , Ishida J , von Haehling S , Anker SD , Springer J . Nutrition in cachexia: from bench to bedside. J Cachexia Sarcopenia Muscle 2016; 7: 107–109. 27030816 10.1002/jcsm.12111 PMC4788973

[41] Loncar G , Springer J , Anker M , Doehner W , Lainscak M . Cardiac cachexia: hic et nunc. J Cachexia Sarcopenia Muscle 2016; 7: 246–260. 27386168 10.1002/jcsm.12118 PMC4929818

[42] Malmstrom TK , Miller DK , Simonsick EM , Ferrucci L , Morley J . SARC‐F: a symptom score to predict persons with sarcopenia at risk for poor functional outcomes. J Cachexia Sarcopenia Muscle 2016; 7: 28–36. 27066316 10.1002/jcsm.12048 PMC4799853

[43] Beaudart C , Biver E , Reginster JY , Rizzoli R , Rolland Y , Bautmans I , Petermans J , Gillain S , Buckinx F , Dardenne N , Bruyère O . Validation of the SarQoL, a specific health‐related quality of life questionnaire for sarcopenia. J Cachexia Sarcopenia Muscle 2017; 8: 238–244. 27897430 10.1002/jcsm.12149 PMC5377391

[44] Scott JS , Martin DS , Ploutz‐Snyder R , Matz T , Caine T , Down M , Hackney K , Buston R , Ryder JW , Ploutz‐Snyder L . Panoramic ultrasound: a novel and valid tool for monitoring change in muscle mass. J Cachexia Sarcopenia Muscle 2017; 8: 475–481. 28052593 10.1002/jcsm.12172 PMC5476852

[45] Nijholt W , Scafoglieri A , Jager‐Wittenaar H , Hobbelen JSM , van der Schans CP . The reliability and validity of ultrasound to quantify muscles in older adults: a systematic review. J Cachexia Sarcopenia Muscle 2017; 8: 702–712. 28703496 10.1002/jcsm.12210 PMC5659048

[46] Siracusa J , Koulmann N , Banzet S . Circulating myomiRs: a new class of biomarkers to monitor skeletal muscle in physiology and medicine. J Cachexia Sarcopenia Muscle 2018; 9: 20–27. 29193905 10.1002/jcsm.12227 PMC5803618

[47] Yang QJ , Zhao JR , Hao J , Li B , Huo Y , Han YL , Wan LL , Li J , Huang J , Lu J , Yang GJ , Guo C . Serum and urine metabolomics study reveals a distinct diagnostic model for cancer cachexia. J Cachexia Sarcopenia Muscle 2018; 9: 71–85. 29152916 10.1002/jcsm.12246 PMC5803608

[48] Loumaye A , Thissen JP . Biomarkers of cancer cachexia. Clin Biochem 2017; 50: 1281–1288. 28739222 10.1016/j.clinbiochem.2017.07.011

[49] Drescher C , Konishi M , Ebner N , Springer J . Loss of muscle mass: current developments in cachexia and sarcopenia focused on biomarkers and treatment. Int J Cardiol 2016; 202: 766–772. 26474466 10.1016/j.ijcard.2015.10.033

[50] Steinbeck L , Ebner N , Valentova M , Bekfani T , Elsner S , Dahinden P , Hettwer S , Scherbakov N , Schefold JC , Sandek A , Springer J , Doehner W , Anker SD , von Haehling S . Detection of muscle wasting in patients with chronic heart failure using C‐terminal agrin fragment: results from the Studies Investigating Co‐morbidities Aggravating Heart Failure (SICA‐HF). Eur J Heart Fail 2015; 17: 1283–1293. 26449626 10.1002/ejhf.400

[51] Scherbakov N , Knops M , Ebner N , Valentova M , Sandek A , Grittner U , Dahinden P , Hettwer S , Schefold JC , von Haehling S , Anker SD , Joebges M , Doehner W . Evaluation of C‐terminal agrin fragment as a marker of muscle wasting in patients after acute stroke during early rehabilitation. J Cachexia Sarcopenia Muscle 2016; 7: 60–67. 27066319 10.1002/jcsm.12068 PMC4799857

[52] Ebner N , von Haehling S . Unlocking the wasting enigma: highlights from the 8th Cachexia Conference. J Cachexia Sarcopenia Muscle 2016; 7: 90–94. 27128291 10.1002/jcsm.12106 PMC4799863

[53] Reihnierse EM , de Jong N , Trappenburg MC , Blauww GJ , Butler‐Browne G , Gapeyeva H , Hogrle JY , McPhee JS , Narici MV , Sipilä S , Stenroth L , van Lummel RC , Pihnappels M , Meskers CGM , Maier AB . Assessment of maximal handgrip strength: how many attempts are needed? J Cachexia Sarcopenia Muscle 2017; 8: 466–474. 28150387 10.1002/jcsm.12181 PMC5476859

[54] Leong DP , Teo KK , Rangarajan S , Kutty VR , Lanas F , Hui C , Quanyong X , Zhenzhen Q , Jinhua T , Noorhassim I . Reference ranges of handgrip strength from 125,462 healthy adults in 21 countries: a prospective urban rural epidemiologic (PURE) study. J Cachexia Sarcopenia Muscle 2016; 7: 535–546. 27104109 10.1002/jcsm.12112 PMC4833755

[55] Kohara K , Okada Y , Ochi M , Ohara M , Nagai T , Tabara Y , Igasa M . Muscle mass decline arterial stiffness, white matter hyperintensity, and cognitive impairment: Japan Shimanami Health Promoting Program study. J Cachexia Sarcopenia Muscle 2017; 8: 557–566. 28371474 10.1002/jcsm.12195 PMC5566649

[56] Fabbri E , Chikles Shaffer N , Gonualez‐Freire M , Shardell MD , Zoli M . Early body composition, but not body mass, is associated with future accelerated decline in muscle quality. J Cachexia Sarcopenia Muscle 2017; 8: 490–499. 28198113 10.1002/jcsm.12183 PMC5476863

[57] Clark AL , Coats AJS , Krum H , Katus HA , Mohacsi P , Salekin D , Schultz MK , Packer M , Anker SD . Effect of beta‐adrenergic blockade with carvedilol on cachexia in severe chronic heart failure: results from the COPERNICUS trial. J Cachexia Sarcopenia Muscle 2017; 8: 549–556. 28244261 10.1002/jcsm.12191 PMC5566644

[58] Foong YI , Chherawala N , Aitken D , Scott D , Winzenberg T , Jones G . Accelerometer‐determined physical activity, muscle mass, and leg strength in community‐dwelling older adults. J Cachexia Sarcopenia Muscle 2016; 7: 275–283. 27239404 10.1002/jcsm.12065 PMC4863829

[59] Lemos Pinto C , Borges Botelho P , Alves Carneiro J , Mota JF . Impact of creatine supplementation in combination with resistance training on lean mass in the elderly. J Cachexia Sarcopenia Muscle 2016; 7: 413–421. 27239423 10.1002/jcsm.12094 PMC4864174

[60] Barbat‐Artigas S , Garnier S , Joffroy S , Riesco E , Sanguignol F , Vellas B , Rolland Y , Andrieu S , Aubertin‐Leheudre M , Mauriège P . Caloric restriction and aerobic exercise in sarcopenic and non‐sarcopenic obese women: an observational and retrospective study. J Cachexia Sarcopenia Muscle 2016; 7: 289–289. 10.1002/jcsm.12075 PMC4867658 27247859

[61] Connolly B , Salisbury L , O'Neill B , Geneen L , Douiri A , Grocott MPW , Hart N , Walsh TS , Blackwood B . Exercise rehabilitation following intensive care unit discharge for recovery from critical illness: executive summary of a Cochrane Collaboration systematic review. J Cachexia Sarcopenia Muscle 2016; 7: 520–526. 27891297 10.1002/jcsm.12146 PMC5114628

[62] Chan DC , Tsou HH , Chang CB , Yang RS , Tsauo JY , Chen CY , Hsiao CF , Hsu YT , Chen CH , Chang SF , Hsiung CA , Kuo KN . Integrated care for geriatric frailty and sarcopenia: a randomized control trial. J Cachexia Sarcopenia Muscle 2017; 8: 78–88. 27897406 10.1002/jcsm.12132 PMC5326822

[63] Snijders T , Nederveen JP , Hoanisse S , Leenders M , Verdijk LB , van Loon LJC , Parise G . Muscle fibre capillarization is a critical factor in muscle fibre hypertrophy during resistance exercise training in older men. J Cachexia Sarcopenia Muscle 2017; 8: 267–276. 27897408 10.1002/jcsm.12137 PMC5377411

[64] Barmouin Y , McPhee JS , Butler‐Browne G , Bosutti A , De Vito G , Jones DA , Marici M , Behin A , Hogrel JY , Degens H . Coupling between skeletal muscle fiber size and capillarization is maintained during healthy aging. J Cachexia Sarcopenia Muscle 2017; 8: 647–659. 28382740 10.1002/jcsm.12194 PMC5566646

[65] Verzola D , Bonanni A , Sofia A , Montecucco F , D'Amato E , Cademartori V , Parodi EL , Vlazzi F , Venturelli C , Brunori G , Garibotto G . Toll‐like receptor 4 signalling mediates inflammation in skeletal muscle of patients with chronic kidney disease. J Cachexia Sarcopenia Muscle 2017; 8: 131–144. 27897392 10.1002/jcsm.12129 PMC5326826

[66] Yu R , Chen JA , Xu J , Cao J , Wang Y , Thomas SS , Hu Z . Suppression of muscle wasting by the plant‐derived compound ursolic acid in a model of chronic kidney disease. J Cachexia Sarcopenia Muscle 2017; 8: 327–341. 27897418 10.1002/jcsm.12162 PMC5377392

[67] Molina P , Carrero JJ , Bover J , Chauveau P , Mazzaferro S , Urena Torres P , for ERN and CKD‐MBD, ERA‐EDTA . Vitamin D, a modulator of musculoskeletal health in chronic kidney disease. J Cachexia Sarcopenia Muscle 2017; 8: 686–701. 28675610 10.1002/jcsm.12218 PMC5659055

[68] Enoki A , Watanabe H , Arake R , Fujimura R , Ishiodori K , Imafuku T , Nishida K , Sugimoto R , Nagao S , Miyamura S , Ishima Y , Tanaka M , Matsushita K , Komaba H , Fukagawa M , Otagiri M , Maruyama T . Potential therapeutic interventions for chronic kidney disease‐associated sarcopenia via indoxyl sulfate‐induced mitochondrial dysfunction. J Cachexia Sarcopenia Muscle 2017; 8: 735–747. 28608457 10.1002/jcsm.12202 PMC5659061

[69] Kittiskulnam P , Carreor JJ , Chertow GM , Kaysen GA , Delgado C , Johansen KL . Sarcopenia among patients receiving hemodialysis: weighing the evidence. J Cachexia Sarcopenia Muscle 2017; 8: 57–68. 27897415 10.1002/jcsm.12130 PMC5326818

[70] Sanders KJC , Kneppers AEM , van de Bool C , Langen RCJ , Schols AMWJ . Cachexia in chronic obstructive pulmonary disease: new insights and therapeutic perspective. J Cachexia Sarcopenia Muscle 2016; 7: 5–22. 27066314 10.1002/jcsm.12062 PMC4799856

[71] van de Bool C , Rutten EPA , van Helvoort A , Franssen FME , Wouters EFM , Schols AMWJ . A randomized clinical trial investigating the efficacy of targeted nutrition as adjunct to exercise training in COPD. J Cachexia Sarcopenia Muscle 2017; 8: 748–758. 28608438 10.1002/jcsm.12219 PMC5659064

